# EVA1A Plays an Important Role by Regulating Autophagy in Physiological and Pathological Processes

**DOI:** 10.3390/ijms22126181

**Published:** 2021-06-08

**Authors:** Shizhen Zhao, Honggang Wang

**Affiliations:** Henan International Joint Laboratory of Nuclear Protein Regulation, School of Basic Medical Sciences, Henan University, Kaifeng 475000, China; 40060002@vip.henu.edu.cn

**Keywords:** EVA1A, autophagy, apoptosis, tumor, liver injury

## Abstract

Eva-1 homolog A (EVA1A) is regarded as TMEM166 (transmembrane protein 166) or FAM176A (family with sequence similarity 176) and a lysosome and endoplasmic reticulum-associated protein involved in regulating autophagy and apoptosis. EVA1A regulates embryonic neurogenesis, cardiac remodeling, islet alpha-cell functions, acute liver failure, and hepatitis B virus replication. However, the related mechanisms are not fully clear. Autophagy is a process in which cells transfer pathogens, abnormal proteins and organelles to lysosomes for degradation. It plays an important role in various physiological and pathological processes, including cancer, aging, neurodegeneration, infection, heart disease, development, cell differentiation and nutritional starvation. Recently, there are many studies on the important role of EVA1A in many physiological and pathological processes by regulating autophagy. However, the related molecular mechanisms need further study. Therefore, we summarize the above-mentioned researches about the role of EVA1A in physiological and pathological processes through regulating autophagy in order to provide theoretical basis for future researches.

## 1. Introduction

Eva-1 homolog A (EVA1A), also known as FAM176A (family with sequence similarity 176) or TMEM166 (transmembrane protein 166), is a novel human gene and a lysosome and endoplasmic reticulum-associated protein that regulates autophagy and apoptosis [1,2,3]. It is conserved in humans, chimpanzees, rats, dogs and mice, suggesting that it may have a vital role in vertebrate animals [4]. The expression of EVA1A is cell and tissue specific and is notably decreased in cancer [2,5,6,7]. EVA1A overexpression suppresses tumors by regulating autophagy and apoptosis [3,8]. Studies indicate that EVA1A promotes the recruitment of ATG12–ATG5/ATG16L1 complex to the autophagic membrane through the interaction of its C-terminal with ATG16L1, and induces autophagosome formation and programmed cell death [9]. Other studies show that EVA1A plays an important role in promoting C/EBPα-mediated autophagy [10].

**Figure 1:**
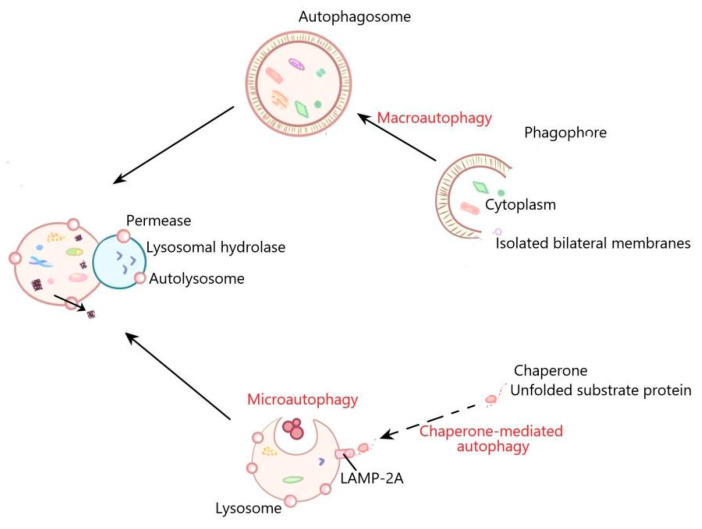
The general process of macroautophagy, microautophagy, and chaperone-mediated autophagy. In the process of macroautophagy, the contents are encapsulated by a bilayer membrane structure to form autophagosomes. Autophagosomes then fuse with lysosomes to form autolysosomes, in which the separated contents are usually degraded for recycling. Microautophagy refers to the direct invagination of lysosomal membrane, which then encapsulates cell contents. In the process of chaperone mediated autophagy, the cytoplasmic proteins are transported to lysosomal chambers after binding with chaperones, and then digested by lysosomal enzymes.

Autophagy is a cellular recycling system that exists in almost all types of eukaryotes. The system is composed of a variety of proteins. The function of these proteins is to form autophagosomes to transport the intracellular cargo to lysosomes for degradation [11]. Autophagy is crucial for cell homeostasis by removing cellular molecules such as protein aggregates and damaged organelles with lysosomal digestion [12,13]. At present, autophagy can be divided into three types: macroautophagy, microautophagy and chaperone-mediated autophagy [14]. Macroautophagy, which is known as bulk autophagy, promotes the formation of autophagosomes, which are double membrane vesicles used to isolate part of the cytoplasm. Autophagosomes then fuse with lysosomes to form autolysosomes, in which the separated cytoplasm is usually degraded for recycling [13,15]. Microautophagy refers to the entry of cytoplasmic contents into lysosomes through invagination or deformation of the lysosomal membrane [16]. Chaperone-mediated autophagy is a kind of selective autophagy, in which intracellular proteins are bound to chaperones and then transported to lysosomal chambers for degradation [17,18] (Figure 1). LC3, Beclin 1, p62 and ATG12-ATG5 are important autophagy markers. LC3 includes LC3-I and LC3-II. LC3-I is lipidated in an ubiquitin-like reaction and is essential for efficient autophagosome formation. LC3-II is a crucial component for efficient autophagosome formation. When the autophagosome formation is increased or the autophagosome degradation is reduced, the level of LC3-II is increased [19]. Becline-1 regulates autophagosomes formation [20,21]. p62 can connect LC3 and ubiquitinated substrate, then be integrated into autophagosomes and degraded in lysosome. When autophagy is activated, autophagosomes fuse with lysosome, p62 is degraded by lysosomal enzymes, and the level of p62 is decreased; when autophagy is inhibited, the p62 level is increased [22]. ATG12 forms a complex with ATG5, which plays an important role in the autophagosome formation and ATG8/LC3 lipidation. Deletion of ATG12-ATG5 leads to autophagy defects [23,24]. Under physiological conditions, autophagy is usually maintained at the basic level and reduces the accumulation of damaged protein aggregates and organelles [25]. Autophagy is a “double-edged sword”. Under the induction of pathological factors including ischemia, pathogenic infection, protein misfolding, hypoxia and nutritional deficiency, etc., the enhanced autophagy can clear the pathological factors in cells to maintain cell survival, suggesting that autophagy is “beneficial” [26]. However, autophagy can cause cell death if it persists at a high level for a long time, suggesting that autophagy is “detrimental” [27,28]. Autophagy has been proven to be involved in physiological processes including adaptation to starvation, cell differentiation and development, degradation of abnormal structures, conversion of redundant or damaged organelles, tumor suppression, innate and adaptive immunity, prolongation of life span and cell death [29]. Dysfunctional autophagy is associated with a variety of diseases, such as cancer, infection, autoimmune and neurodegenerative diseases [30]. Recently, it has been reported that EVA1A plays an important role through influencing autophagy in many physiological and pathological processes. Here, we reviewed the current knowledge about the role of EVA1A in regulating autophagy to provide references for future research.

## 2. EVA1A Plays an Important Role by Regulating Autophagy in Tumor

**Figure 2:**
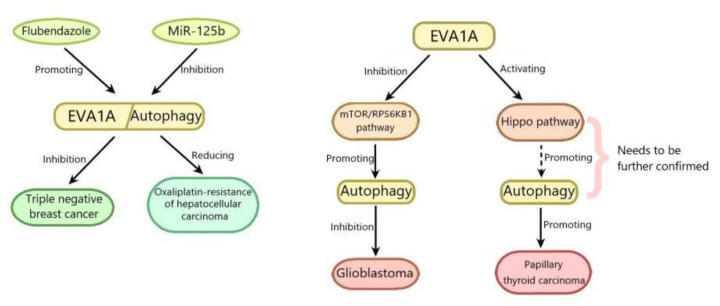
EVA1A plays an important role by regulating autophagy in the regulation of tumors.

Hepatocellular carcinoma (HCC) accounts for about 90% of all cases of primary liver cancer, which is the second leading cause of cancer-related death in the world. The main risk factors of HCC are well known, including hepatitis B and hepatitis C virus infection, alcohol intake and fungal metabolite aflatoxin B1 intake. In recent years, the knowledge about other risk factors such as nonalcoholic steatohepatitis is increasing [31]. HCC is most often diagnosed as advanced stage. At this time, the resection rate is low and the recurrence rate is high [32,33]. Oxaliplatin (a platinum compound)-based chemotherapy has recently been proved to be an effective method for the treatment of advanced liver cancer; however, the severe development of drug resistance has greatly inhibited its efficacy [34]. Therefore, it is very important to find a way to inhibit oxaliplatin resistance for the treatment of HCC. MicroRNAs (miRNAs) are highly conserved and small non-coding RNAs that silence the target gene by binding to its 3′-untranslated region (UTR). MiR-125b is the first mammalian ortholog of mirna-lin-4 found in Caenorhabditis elegans and regulates many physiological and pathological processes such as HCC [35]. MiR-125b has been reported to inhibit HCC through suppressing the proliferation, invasion and tumorigenicity of hepatoma cells, and is considered as a biomarker to predict the prognosis of patients with HCC [36,37]. However, its role in cancer drug resistance has yet to be confirmed. The results of Wei Wei Ren and colleagues showed that the expression of miR-125b in oxaliplatin-resistant HCC tissues and cell lines decreased. Compared with oxaliplatin-resistant HCC cell lines, the overexpression of miR-125b in sensitive cells reduced oxaliplatin resistance through suppressing cell proliferation, migration and epithelial-mesenchymaltransition (EMT), while treatment with anti-miR-125b abolished the changes, indicating that miR-125b might inhibit oxaliplatin resistance. The results of luciferase reporter gene assay revealed that miR-125b binded to the EVA1A 3′-UTR and inhibited its expression, suggesting that EVA1A is the direct target gene of miR-125b. The in depth researches showed that EVA1A expression was increased in oxaliplatin-resistant HCC tissues, and its ectopic expression partially promoted autophagy and reversed the inhibitory effect of miR-125b on the growth of oxaliplatin resistant cell lines and xenografts. The results of Western blot showed that miR-125b reversed the increase of LC3-II/LC3-1 ratio and beclin-1 expression and the reduction of p62 induced by EVA1A, indicating that miR-125b suppressed autophagy induced by EVA1A. Taken together, miR-125b reduces oxaliplatin resistance by decreasing EVA1A-mediated autophagy, which provides a possible way to overcome chemotherapy resistance by regulating the expression of miR-125b or EVA1A [38] (Figure 2). The role of miR-125b in tumors has been reported to be related to the p53 signaling pathway [39,40]. Therefore, whether the p53 signaling pathway is involved in the regulation of EVA1A by miR-125b needs to be elucidated. It can be seen from the above that the enhancing of the EVA1A-regulated autophagy can increase the drug resistance of HCC and reduce the chemotherapy effect of HCC. Similarly, the enhancing of the EVA1A-regulated autophagy can directly promote the growth of tumor cells. Papillary thyroid carcinoma (PTC) is the most common endocrine malignant tumor, accounting for 85% of all well differentiated follicular thyroid carcinoma and occurring mainly in females. Its 10-year survival rate is about 93% [41,42]. Most PTC patients have a good prognosis after surgical resection combined with radioiodine therapy. However, the incidence rate and mortality of advanced papillary thyroid cancer have been increasing in recent years [43]. In order to study the pathogenesis of PTC, Bang-Yi Lin and colleagues committed a series of experiments and found that the EVA1A expression level in tumor tissues of 43 patients with PTC was significantly higher than that in adjacent normal tissues. The logistic regression analysis of the TCGA data set showed that EVA1A was an independent hazard factor for PTC. The in depth study revealed that EVA1A knockdown by siRNA inhibited PTC cells proliferation and colony formation, invasion and migration, and promoted PTC cells apoptosis. EVA1A knockdown by siRNA reduced the expression of N-cadherin, vimentin, Bcl-xL Yes-associated protein (YAP) and transcriptional co-activator with PDZ-binding motif (TAZ), and increased bax expression, suggesting that EVA1A knockdown promoted apoptosis and inhibited epithelial-mesenchymal transition (EMT) progression via inhibiting Hippo pathway. Collectively, it can be deduced that EVA1A promotes the progression and EMT of PTC through activating Hippo pathway (Hippo pathway can limit the growth of adult tissues and regulate the cell proliferation, differentiation and migration in developing organ), which needs further confirmation in vivo [44] (Figure 2). The molecular mechanisms of EVA1A in PTC are very complex, and whether EVA1A promotes the progression and EMT of PTC by activating autophagy remains to be elucidated. With the deepening of research, EVA1A may be used as an effective index for diagnosis and treatment of PTC.

Contrary to the above, the enhanced EVA1A-regulated autophagy can inhibit tumors. Glioblastoma (GBM) is the most common malignant brain tumor in the central nervous system. There is no effective treatment at present [45,46]. Therefore, it is particularly important to explore the molecular mechanism of this disease. It has been reported that autophagy dysregulation can cause cells’ malignant transformation [47], leading to many cancers, including GBM [48]. Xue Shen et al. overexpressed EVA1A in glioblastoma (GBM) cell line by transfection with recombinant adenovirus 5-EVA1A vector (Ad5-EVA1A) and studied its antitumor activity in vitro. The results showed that EVA1A overexpression suppressed the proliferation of GBM cells. The mechanism research revealed that EVA1A overexpression promoted autophagy through inducing autophagosome formation, reducing autophagosome clearance, increasing the levels of LC3B-II and decreasing the levels of p62 [8]. The mammalian target of rapamycin(mTOR)/ribosomal protein S6 kinase B1(RPS6KB1) pathway has been reported to be involved in the regulation of autophagy [49]. The mTOR regulates the initiation, process and termination of autophagy, through modulating the activity of the vacuolar protein sorting 34 (VPS34) complex, unc51-like kinase 1 (ULK1) complex, and the intracellular distribution of TFEB/TFE3 and proto-lysosome tubule reformation [50]. EVA1A overexpression notably reduced the phosphorylation levels of mTOR and RPS6KB1, suggesting that EVA1A overexpression inhibited the mTOR/RPS6KB1 pathway. MHY1485, which could activate mTOR and suppressed lysosome fusion, increased the phosphorylation levels of mTOR and RPS6KB1 suppressed by EVA1A overexpression, and abolished the effects of EVA1A overexpression on LC3B-II/p62, suggesting that EVA1A overexpression induced autophagy through inhibiting mTOR/RPS6KB1 pathway. In addition to autophagy, EVA1A overexpression could induce apoptosis at 48 h after transfection with Ad5-EVA1A, but not at the early stage (24 h). Collectively, it can be deduced that EVA1A suppresses GBM cells proliferation by promoting autophagy through inhibiting the mTOR/RPS6KB1 pathway, which needs further confirmation in vivo [8] (Figure 2). Autophagy and apoptosis have been reported to be related with cancer [51,52], so the interplay between autophagy and apoptosis induced by EVA1A in GBM needs to be elucidated. Similarly, EVA1A-regulated autophagy can suppress breast cancer. Triple negative breast cancer (TNBC) is a subtype of breast cancer, which is lack of estrogen receptor (ER), progesterone receptor (PR) or human epidermal growth factor receptor 2 (HER-2). TNBC is characterized by strong invasion, high metastatic potential, easy recurrence and poor prognosis and is not sensitive to endocrine therapy or HER2 therapy, therefore, there is no effective treatment for TNBC [53,54]. Flubendazole is a broad-spectrum anthelmintic drug of the benzimidazole group and reported to be effective in several malignancies including breast carcinoma, colorectal carcinoma and neuroblastoma [55]. The results of Yongqi Zhen et al. showed that after treatment with flubendazole, the proliferation of TNBC cells was notably suppressed and the colony formation was decreased. Similar results were obtained in a xenograft tumor model of mice in vitro. The mechanism research revealed that flubendazole increased apoptosis of TNBC cells through upregulating the expression levels of cleaved caspase 3 and Bax, and downregulating Bcl-2 expression. Flubendazole also promoted autophagy by increasing the levels of LC3-II/LC3-I and Beclin-1, and promoting p62 degradation. Moreover, fluendazole could promote the formation of autolysosomes and autophagosomes. However, 3-MA (an autophgy inhibitor by interrupting autophagosome formation) suppressed autophagy and partially reversed flubendazole-induced apoptosis, indicating that autophagy mediated flubendazole induced apoptosis of TNBC cells. Flubendazole inhibits TNBC metastasis via increasing E-cadherin expression and decreasing MMP-2 expression in vivo and in vitro, which was alleviated by combined flubendazole treatment with 3-MA or ATG5 knockdown, suggesting that autophagy mediated the inhibition of TNBC metastasis by flubendazole. EVA1A expression was decreased in tumor tissues of TNBC and increased in flubendazole-treated TNBC in vivo and in vitro. EVA1A knockdown with siRNA alleviated the inhibition of autophagy, apoptosis and metastasis of TNBC by flubendazole, indicating that flubendazole promoted autophagy and inhibited proliferation and migration of TNBC cells by upregulating EVA1A. Furthermore, EVA1A overexpression could not promote the LC3 aggregation in ATG5 knockdown TNBC cells, suggesting that EVA1A-induced autophagy depended on ATG5. In addition, EVA1A overexpression also notably suppressed cell growth and proliferation in TNBC cells, which was attenuated in ATG5-depleted TNBC cells. These above indicated that flubendazole inhibited apoptosis and metastasis of TNBC by promoting autophagy via ATG5. The study on the combination of flubendazole and EVA1A showed that the point mutation of Thr113 in EVA1A mitigated the inhibition of autophagy, apoptosis and metastasis of TNBC by flubendazole, indicating that flubendazole might affect EVA1A by binding Thr113 in EVA1A. Collectively, flubendazole induced autophagy-mediated death of TNBC cells by targeting EVA1A, thus inhibiting tumor proliferation and migration [56] (Figure 2). It has been reported that flubendazole can promote autophagy through Atg4B, which is related with ATG8/LC3 conjugation in autophagosome formation [57]. Therefore, whether EVA1A promotes autophagy by activating ATG4B and then improves TNBC is worthy of further study.

## 3. EVA1A Plays an Important Role by Regulating Autophagy in Liver Injury

Acute liver failure (ALF) is a clinical syndrome characterized by rapid liver destruction, multiple organ failure and cells death [58,59]. Although ALF has been widely studied, its mechanism has not been fully understood. To further study the ALF mechanism, Xin Lin and colleagues constructed an ALF model in mice by co-administration with D-galactosamine (D-GalN) and lipopolysaccharide (LPS) and found that EVA1A expression was reduced at 2 h, increased at 4 h, and then decreased at 6 h. Similar results were found in the expression of autophagy-related proteins such as Atg12-5, Atg16L1, and Beclin1, thus indicating that EVA1A-mediated autophagy might play a role in D-GalN/LPS-induced ALF. The hepatocyte specific deletion of EVA1A aggravated the liver injury of ALF mice, which was characterized by the increased levels of alanine aminotransferase (ALT), aspartate aminotransferase (AST), myeloperoxidase (MPO) and inflammatory cytokines (such as TNF and IL-6), and led to the liver structural disorder in EVA1A^−/−^ mouse livers with ALF. In addition, the reduction of autophagy in EVA1A^−/−^ mouse liver led to a large number of mitochondria swelling in ALF, causing insufficient ATP production or hepatocyte apoptosis. EVA1A overexpression with adeno-associated virus EVA1A (AAV-EVA1A) or autophagy inducer rapamycin can increase the autophagy and improved liver injury of EVA1A^−/−^ mice with ALF. In conclusion, it can be deduced that EVA1A-mediated autophagy improves liver injury in ALF mice by reducing inflammation and apoptosis, suggesting a potential therapeutic role for ALF [60]. It can be seen from the above that EVA1A-mediated autophagy maintain mitochondrial homeostasis to alleviate ALF, the mechanisms of which deserve to be further studied. 

Hepatic ischemia/reperfusion (I/R) injury is a severe complication of hypovolemic shock, hepatectomy and liver transplantation, which seriously affects the prognosis of patients [61]. Kupffer cells (KCs) play an important role in hepatic I/R injury [62]. Ziyi Wang et al. found that the expression level of EVA1A was upregulated with the activation of inflammation in mouse liver I/R injury model. The clearance of KCs by chlorophosphonic acid liposome aggravated inflammatory reaction and liver injury, and inhibited the increase of EVA1A expression in mouse liver I/R injury model, indicating that in the stage of inflammatory activation after I/R, the expression of EVA1A mainly in KCs increased, and KCs might play the role of anti-inflammatory and promoting repair in the process of hepatic I/R. injury. The mechanism researches revealed that rapamycin (an autophagy activator) pre-treatment could mitigate inflammatory injury of liver by inhibiting NLRP3 inflammasome activation and reducing the levels of NLRP3, IL-1β and IL-18 in KCs with hepatic I/R injury, whereas 3-MA (an autophagy inhibitor) pre-treatment reversed the effects. Furthermore, the suppression of NLRP3 with MCC950 (a NLRP3 inhibitor) decreased the levels of IL-1β and IL-18 in KCs with hepatic I/R injury. Collectively, it can be deduced that autophagy promotion inhibits NLRP3 inflammasome-mediated inflammation. Moreover, knockdown of EVA1A by siRNA in KCs aggravated liver inflammatory injury through enhancing NLRP3 inflammasome activation and inhibiting autophagy by reducing the autophagosome formation. SiATG5/ATG12 or siATG7 inhibited autophagy activation induced by EVA1A overexpression, while the suppression of Beclin1-vps34 pathway by Vps34 inhibitor PIK-III and siBECN1 could not reverse EVA1A-induced autophagy, suggesting that EVA1A upregulated autophagy through ATG5/ATG12 rather than Beclin1-vps34 pathway. In conclusion, EVA1A improves hepatic I/R injury through inhibiting NLRP3 activation by inducing autophagy in KCs, suggesting that EVA1A may be a drug target for clinical treatment of hepatic I/R injury [63]. From the above, it can be seen that EVA1A can inhibit the liver inflammatory response mediated by NLRP3 inflammasome. Therefore, whether EVA1A has anti-inflammatory function in other tissues or diseases and its anti-inflammatory mechanism are worthy of further study.

## 4. EVA1A Plays an Important Role by Regulating Autophagy in Myocardial Remodeling

Many heart diseases, such as ischemic disease, valvular disease and hypertension, lead to cardiac remodeling and eventually result in heart failure [64]. Cardiac remodeling is considered to be a major determinant of disease progression and outcome in heart failure (HF), and plays a vital role in discovering improved treatment for HF with reduced ejection fraction [65]. To elucidate the mechanisms of cardiac protection against remodeling, Shu Zhang et al. committed a series of experiments and found that the absence of EVA1A in the adult mice heart promoted myocardial fibrosis, induced myocardial hypertrophy, thereby resulting in cardiomyopathy. Further studies showed that this effect was related to the impaired autophagy and increased apoptosis in the heart of EVA1A knockout mice. Furthermore, the knockout of EVA1A activated mTOR pathway by increasing the phosphorylation levels of mTOR, while rapamycin (a specific inhibitor of mTOR) alleviated EVA1A KO effects on mice myocardial remodeling and autophagy, suggesting that EVA1A exerted myocardial protection by inhibiting mTOR pathway. Moreover, the structural disorder of sarcomere, maladjustment and aggregation of mitochondria after EVA1A gene knockout led to insufficient ATP production. In conclusion, the results suggested that EVA1A ameliorated cardiac remodeling through inhibiting cardiac hypertrophy and fibrosis by promoting autophagy via inhibiting mTOR pathway [1]. Further studies are needed to study the possibility of EVA1A as a therapeutic target for HF. It has been reported that myocardial fibrosis is related to oxidative stress [66], so whether EVA1A can protect myocardium through antioxidant stress remains to be studied.

## 5. EVA1A Plays an Important Role by Regulating Autophagy in Atherosclerosis

Atherosclerosis (AS) is a chronic inflammatory disease with autoimmune components, which is the main cause of cardiovascular diseases [67,68]. The gradual accumulation of atherosclerotic plaque blocks blood flow to lead to atherosclerosis, resulting in heart attack or stroke [69]. At present, the rapid re-endothelialization is an effective method to inhibit AS. In order to elucidate the role of EVA1A in re-endothelialization of injured arteries, Jingxuan Li et al. performed many experiments and found that the re-endothelialization was inhibited in endothelial cells (ECs) of wire-injured EVA1A^−/−^ mice carotid arteries or ECs-specific EVA1A knockout mice, suggesting that EVA1A deletion suppressed re-endothelialization of wire-injured mice carotid arteries. Furthermore, EVA1A deletion also suppressed re-endothelialization in AS-prone mice (EVA1A^−/−^ApoE^−/−^ mice). EVA1A overexpression by Ad5-EVA1A increased ECs repair by promoting ECs migration in vivo and vitro. The results of proteomic analysis showed that Arpc1b is the downstream target protein of EVA1A in ECs. The expression of Arpc1b and its related proteins, Cdc42 and Rac1 was decreased by si-EVA1A and increased by Ad5-EVA1A in EC, and si-Arpc1b inhibited EVA1A-induced migration in EC, suggesting that EVA1A promoted EC migration through the Rac1/Cdc42/Arpc1b pathway. Moreover, EVA1A deletion accelerated the development of AS, evidenced by the increased lesion formation and neointimal hyperplasia. Collectively, it can be deduced that EVA1A improves AS through increasing re-endothelialization of injured arteries by promoting ECs migration via Rac1/Cdc42/Arpc1b, which suggests that EVA1A is a potential therapeutic target for AS [70]. The above inference need to be further verified by the inhibitor of the ECs migration. It has been reported that vascular ECs dysfunction caused by autophagy is considered to be the earliest stage of AS, which leads to the formation of atherosclerotic plaque and subsequent cardiovascular complications [71,72,73]. Autophagy is related to migration and proliferation of ECs [74], and EVA1A can upregulate autophagy [9]. Therefore, it can be deduced that EVA1A may regulate ECs function through autophagy, which needs further study.

## 6. EVA1A Plays an Important Role by Regulating Autophagy in Embryonic Neurogenesis

Neurogenesis is the basic process of human highly evolved central nervous system (CNS). It is carried out by the differentiation of particular neural precursor cells in the neurogenic niche [75]. Embryonic neurogenesis is crucial for the maintenance of neural stem cells (NSCs), so it is very vital for the development and function of brain [76]. The results of Mengtao Li and colleagues demonstrated that the levels of autophagy and EVA1A were significantly increased during the neuron differentiation. The experiment with EVA1A^−/−^ mice showed that EVA1A depletion inhibited NSCs self-renewal in vivo and in vitro, which is not caused by apoptosis. The differentiation of NSCs was also suppressed by EVA1A deletion, whereas EVA1A overexpression had the opposite effects. Moreover, EVA1A depletion downregulated autophagy by decreasing the levels of ATG5 and LC3B-II and increasing p62 level in vivo and in vitro. The levels of p-mTOR, p-RPS6KB1, p-EIF4EBP1 PIK3CA and AKT were upregulated, and the phosphorylation levels of TSC2 was downregulated in EVA1A^−/−^ cortex or EVA1A^−/−^ NSCs, suggesting that EVA1A depletion activated the PIK3CA-AKT pathway, resulting the activation of mTOR. As mTOR has been known to negatively regulate autophagy, thus the PIK3CA-AKT pathway-induced mTOR inhibited autophagy. While EVA1A overexpression with Ad5-EVA1A in EVA1A^−/−^ NSCs inhibited phosphatidylinositol 3-kinase alpha/protein kinase B-mammalian target of rapamycin(PIK3CA/AKT-MTOR)pathway and increased autophagy. Enhancing autophagy by rapamycin (an autophagy activator) could reversed the inhibitory effects of EVA1A depletion on NSCs self-renewal and differentiation. Furthermore, Adding methylpyruvate during the differentiation of neural stem cells can save the defects of embryonic neurogenesis induced by EVA1A deletion, suggesting that energy utilization is an important factor in embryonic neurogenesis. In conclusion, EVA1A promotes embryonic neurogenesis by upregulating autophagy via inhibiting the PIK3CA-AKT/mTOR pathway [77]. The precise mechanism of EVA1A regulating the PIK3CA-AKT/mTOR pathway needs to be further elucidated. Autophagy has been reported to provide energy for neurogenesis [78]. Therefore, it can be deduced that EVA1A promotes autophagy, which can provide energy for NSCs self-renewal and differentiation. EVA1A-regulated autophagy may be a potential target for the treatment of neurodevelopmental disorders.

## 7. Conclusions

EVA1A is a new protein discovered in recent years, which can regulate autophagy and apoptosis. This review summarized the current research on the role of eva-1 in regulating autophagy in a variety of pathophysiological processes: (1) MiR-125b reduces oxaliplatin resistance by decreasing EVA1A-mediated autophagy; (2) EVA1A suppresses glioblastoma cells proliferation by promoting autophagy through inhibiting mTOR/RPS6KB1 pathway; (3) EVA1A promotes the progression and EMT of PTC through activating autophagy via hippo pathway, which needs to be further conformed; (4) Flubendazole induced autophagy-mediated death of TNBC cells by promoting EVA1A-regulated autophagy, thus inhibiting tumor proliferation and migration; (5) EVA1A-mediated autophagy improves ALF by maintaining mitochondrial homeostasis; (6) EVA1A improves hepatic I/R injury through inhibiting NLRP3 activation by inducing autophagy via inhibiting mTOR pathway in KCs; (7) EVA1A ameliorates cardiac remodeling through inhibiting cardiac hypertrophy and fibrosis by promoting autophagy; (8) EVA1A may improve atherosclerosis by regulating ECs function through promoting autophagy; (9) EVA1A promotes embryonic neurogenesis by upregulating autophagy via PIK3CA-AKT/mTOR pathway (Table 1). It can be seen from the above that enhancing the EVA1A-regulated autophagy can inhibit tumor, and on the contrary, the EVA1A-regulated autophagy can promote tumors, which may be due to the different basic expression levels of EVA1A and autophagy in different tumor tissues, or the different roles of EVA1A in different stages of tumors. At present, in general, EVA1A and its regulated autophagy are beneficial to human health in most cases.

The mechanisms of EVA1A regulating autophagy and apoptosis are still to be elucidated, such as how EVA1A up-regulates autophagy by activating ATG4B. The upstream and downstream agents of EVA1A also need to be studied. The signaling pathways involved in the role of EVA1A in various physiological and pathological processes also need to be further studied. Moreover, EVA1A can inhibit NLRP3 inflammasome to alleviate hepatic ischemia-reperfusion injury. Therefore, whether EVA1A can play an important anti-inflammatory role by inhibiting NLRP3 inflammasome may be a question worthy of study.

In addition to the above biological functions, EVA1A should have other biological effects. With the further research, more functions of EVA1A can be found. In the future, EVA1A is likely to become an important substance to improve a variety of diseases.

## Figures and Tables

**Table 1:** The effects of EVA1A-mediated autophagy in different physiological and pathological processes.

The Name of Disease/Pathological Process	State of EVA1A (Autophagy)	Effects
Oxaliplatin-resistant hepatocellular carcinoma	Increasing	MiR-125b reduces oxaliplatin resistance by decreasing EVA1A-mediated autophagy
Glioblastoma	Inhibition	EVA1A suppresses glioblastoma cells proliferation by promoting autophagy through inhibiting mTOR/RPS6KB1 pathway
Papillary thyroid carcinoma (PTC)	Increasing	EVA1A promotes the progression and epithelial-mesenchymal transition of PTC through activating hippo pathway
Triple negative breast cancer (TNBC)	Inhibition	Flubendazole induced autophagy-mediated death of TNBC cells by promoting EVA1A-regulated autophagy
Acute liver failure (ALF)	Inhibition	EVA1A-mediated autophagy improves ALF by maintaining mitochondrial homeostasis
Hepatic ischemia/reperfusion	Inhibition	EVA1A improves hepatic I/R injury through inhibiting NLRP3 activation by inducing autophagy in KCs
Cardiac remodeling	Inhibition	EVA1A ameliorates Cardiac remodeling through inhibiting cardiac hypertrophy and fibrosis by promoting autophagy via inhibiting mTOR pathway
Atherosclerosis	Inhibition	EVA1A may improves atherosclerosis by regulating EC function through promoting autophagy
Embryonic neurogenesis	Inhibition	EVA1A promotes Embryonic Neurogenesis by upregulating Autophagy via inhibiting PIK3CA-AKT/mTOR pathway

EVA1A: Eva-1 homolog A; mTOR/RPS6KB1: mammalian target of rapamycin/ribosomal protein S6 kinase B1; KCs:Kupffer cells; EC: endothelial cells;PIK3CA-AKT/mTOR:phosphatidylinositol 3-kinase alpha/protein kinase B-mammalian target of rapamycin.
